# Effect of Modified Magnesium Oxide on the Properties of Magnesium Phosphate Cement under a Negative Temperature Environment

**DOI:** 10.3390/ma15249047

**Published:** 2022-12-18

**Authors:** Xuanzhang Luo, Zhenyu Lai, Zhi Liu, Rui Xiao, Jiawei Chen, Zhongyuan Lu, Shuzhen Lv, Jin Wang

**Affiliations:** State Key Laboratory of Environmental-Friendly Energy Materials, School of Materials Science and Engineering, Southwest University of Science and Technology, Mianyang 621010, China

**Keywords:** magnesium oxide, surface modification, magnesium phosphate cement, negative-temperature environment, working performance, mechanical properties

## Abstract

As a rapid repair material, magnesium phosphate cement (MPC) can be used under various environmental temperature conditions, but different temperatures significantly impact its strength and working performance. In this study, based on the surface modification of magnesium oxide, the working and mechanical properties of samples were investigated at an ambient temperature of −5 °C, and the hydration properties and microstructure of MPC were investigated using X-ray diffraction (XRD), thermogravimetric analysis (TG), mercury-in-pressure (MIP), and scanning electron microscopy (SEM). The results show that the modified magnesium oxide at a negative temperature prolongs the setting time of MPC from 10 min to more than 30 min, and fluidity can still be maintained or increased after half an hour. From 1 d to 28 d, the compressive strength growth rate of the reference group was 257.0% compared to 723.8% for the 10 wt% water-glass-modified MgO sample. K-struvite transformed from a blocky growth to a needle-like growth with the modified sample filling the pores and cracks inside the matrix. Compared with the unmodified sample, MPC’s porosity decreased from 9.62% to 9.23% for 10 wt% water-glass-modified MgO. Therefore, the surface modification of magnesium oxide not only prolonged the setting time but also further benefited mechanical performance, which provides the prerequisites for MPC construction in negative-temperature environments.

## 1. Introduction

Magnesium phosphate cement (MPC) is widely used as the rapid repair material for airport runways, bridges, piers, highways, and other infrastructures because of its fast hardening, early strength, high bonding strength, and wide adaptability to many environments, which significantly shortens repair times [1,2,3,4]. Control of the setting time and fluidity is critical for technical application, and it is necessary to rapidly ensure a high strength [5].

Magnesium phosphate cement is composed of dead-burned MgO, soluble phosphate, mineral admixture, and retarder mixed in a particular proportion, and its reaction process is based on an acid–base neutralization reaction, and the new air-hardening cementing material is hardened by physical action [6,7,8]. At the early stage of MPC hydration, water molecules are adsorbed on the surface of MgO by coulombic interaction, while cations in the phosphate are also adsorbed on the surface of MgO, which makes MPC have an efficient hydration reaction [9]. The reaction process generates a large amount of heat, which ensures rapid hardening even at negative temperatures [10,11]. Therefore, MPC as a repair material is suitable for use at various ambient temperatures [12]. The addition of magnesium ions to calcium phosphate cements results in a matrix with a higher strength at lower temperatures than at higher temperatures and increased stability of the matrix as the pressure increases [13]. The addition of strontium nitrate, disodium hydrogen phosphate, brushite cement, and hydroxyapatite in calcium phosphate bone cement can improve the strength of the matrix very well but will reduce the cement setting time by more than 30 min, to less than 7 min [14]. Currently, there are related studies on applying MPC to repairing materials. Based on the industry standard for epoxy repair of ballastless tracks, Bing Chen [15] compared it with MPC and found that MPC has the same crack resistance as epoxy. However, MPC has a better compatibility with concrete [16], a lower coefficient of linear expansion, better stability under temperature changes, and a shorter setting time. For damage to concrete structures and hot-mix asphalt pavements, the usual repair materials used are polymer mortar and ordinary silicate cement (OPC) [5,17], but these materials are temperature-sensitive and, thus, degrade. The addition of silica powder to concrete can improve both water and corrosion resistance [18]. Furthermore, the strong temperature spontaneously released by the acid–base reaction process of preparing MPC can cope well with temperature changes and withstand severe weather conditions [19]. The 7 d shear strength of the repaired interface between MPC slurry and silicate cement paste (PCP) reached 5.2 MPa using an MPC simulation for the repair of concrete structures, indicating good bonding between MPC and PCP [20].

Although MPC can achieve rapid hardening in a negative-temperature environment, there are still difficulties regarding its practical application under a negative temperature. When maintained in a negative-temperature environment, MPC tends to fail due to the free water in the capillaries within the matrix while it is freezing and expanding, resulting in damage to the material [21]. In order to solve the problem of mixing water used under a negative temperature for magnesium phosphate cement, Jia [22] tested several types of antifreeze, and it was found that using a mixture with dilute phosphoric acid mixed in water not only enabled MPC to hydrate normally at an ambient temperature of −20 °C, but also enhanced its early strength. The early strength of MPC at a negative temperature is more sensitive to the changes in the boron-to-magnesium ratio (B/M) and water-to-ash ratio (*w*/*s*) than at room temperature, where the intensity decreases with increasing B/M and increases with increasing M/P [10,23]. However, when the temperature is as low as −20 °C, the 2 h compressive strength of MPC is only 3.0 MPa, while the long-term strength of MPC is greatly limited. However, it can be transferred to a normal temperature (20 °C), and maintenance can trigger a second hydration, causing its strength to increase to 67.6% at a normal temperature. Using a small amount of lightly burned magnesium oxide (LBM) instead of heavily burned magnesium oxide can improve the early strength of MPC. When the admixture of LBM reaches 8%, the compressive strength of MPC can exceed 30 MPa when cured at an ambient temperature of −20 °C for 2 h, but it will lose its setting time [24].

There have been some studies applying MPC under negative-temperature conditions; at lower temperatures, MPC has a longer setting time or even stops hydrating due to the external environment. However, in the −5 °C environment, MPC pastes’ setting time is only 10 min, and negative temperature makes early hydration’s limited strength decline, which does not meet the construction requirements. It is still challenging to balance MPC’s setting time and early strength in a negative-temperature (−5°C) environment. Borax is commonly used as retarder at room temperature; in low dosing (B/M < 10%), MPC setting time is often only 8 min or less, while, in high dosing (B/M > 10%), there will be a great loss of MPC strength [25]. The surface modification of MgO using sodium water glass after heat treatment can effectively prolong the setting time of MPC without adversely affecting its later performance [26,27]. There are no studies on the negative temperature of modified MgO. In this study, based on the modified MgO, the MPC slurry was prefabricated at room temperature using ice water and maintained at an ambient temperature of −5 °C. The effects of different sodium water glass contents on MPC’s working and mechanical properties at negative temperatures were investigated, to investigate the applicability of MgO surface modification for MPC at negative temperature and provide a theoretical basis for its application at negative temperature.

## 2. Materials and Experimental Methods

### 2.1. Materials

**Figure 1:**
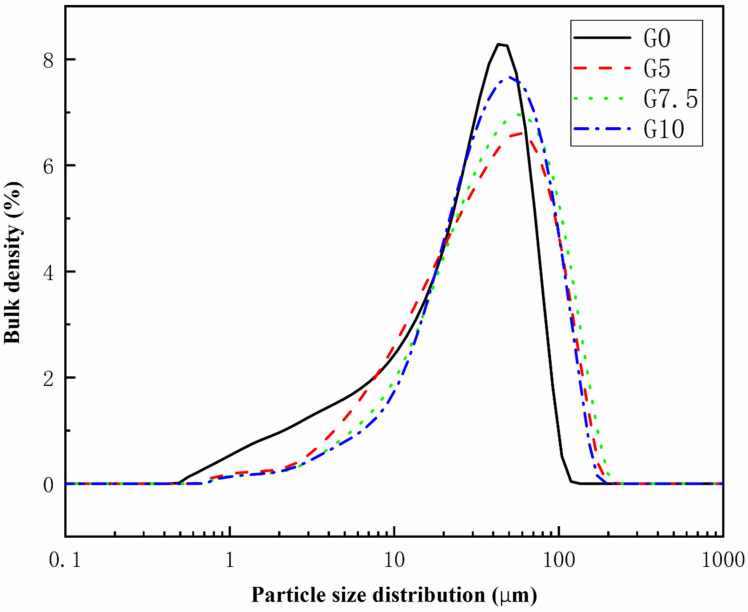
Particle size distribution of different modified MgO.

The dead-burned MgO (abbreviated as M) used in the experiments was provided by China Yancheng Huanai Magnesium Industry Co. It is made of magnesite calcined at 1600 °C with 95% purity and has an average particle size of 29.5µm and a specific surface area of 623.1 m^2^/kg. Chemical composition and particle size distribution were determined by a PANalytical Axios-type X-ray fluorescence spectrometer and a Mastersizer 3000 (Malvern, UK) laser particle size analyzer. The results are shown in Table 1 and Figure 1.

Sodium water glass (Na_2_O·nSiO_2_) is a translucent liquid, with a modulus of 2.8, supplied by Xinjie Chemical Co., Ltd, Mianyang, China. Potassium dihydrogen phosphate (KH_2_PO_4_, KDP, P for abbreviation) and borax (Na_2_B_5_O_7_·10H_2_O, B for abbreviation) are industrially pure products both with a purity of 99.0%. Experimental water used was a mixture of ice and water to ensure the water temperature was 0 °C.

### 2.2. Mixture Design and Specimen Preparation

In order to investigate the workability and mechanical properties of magnesium phosphate cement prepared by changing magnesium oxide with different sodium water glass doping, a sodium water glass is taken as 5%, 7.5%, and 10% of mass ratio for the modification of MgO; the ratio of MgO to KDP is taken as a mass ratio of 3/1. The water–cement ratio is taken as 0.2. Borax is taken as 10% of MgO mass. The specific formula is shown in Table 2.

In the experiment, MgO was first pretreated by taking a certain amount of MgO and sodium water glass in the stirring pot and mixing them well. Then, the mixed material was put into an oven for thermal treatment after the materials were cooled at room temperature, and as-prepared particles were crushed and passed through a 200-mesh fine sieve using a crusher (AK-12A, Wenling Aoli Chinese Medicine Machinery Co., Ltd., Taizhou, China). According to the mix design, M, P, and B were first put into the pot for stirring for 60 s and mixed well, then 0 °C water was added, accompanied by slow stirring for 1 min and 30 s, and then fast stirred for 3 min and 30 s. The prepared MPC slurry was quickly cast into a 40 * 40 * 40 mm mold and immediately vibrated 60 times, and samples were put into a low-temperature test chamber (LRH-250CA, Shanghai Yiheng Scientific Instruments Co., Ltd., China) with the temperature set at −5 °C. Demolding was completed after 2 h, and the obtained samples were wrapped with insulation blankets, put into the low-temperature test chamber again, and maintained at 3 h, 1 d, 3 d, 7 d, and 28 d. Three samples of each component were formed at each age for compressive strength testing.

### 2.3. Analysis and Test Methods

Regarding the workability of MPC, the setting time was determined using a standard Vicameter (China Wuxi Jianyi Instrument & Machinery Co., Ltd., Wuxi, China), according to the Chinese construction industry standard JC/T 2537-2019. Measurements were made every 30 s, from the addition of water to the near-final setting, and the probe was recorded when it did not leave traces on the sample surface. The fluidity of MPC slurry was determined according to the Chinese national standard GB/T 2419-2005. The truncated cone mold was filled with MPC slurry, and then the mold was vertically lifted, so that the slurry flowed freely for 30 s. The diameter of the slurry ground in two mutually perpendicular directions was measured with calipers, and the average value was taken.

Hydration temperature was recorded using an electronic temperature recorder (RC-5+, Jiangsu Jingchuang Electric Co., Ltd., Xuzhou, China): insert the end of the recorder with the metal probe into the middle of the freshly formed sample and place it at −5 °C to record the 3 h hydration exothermic changes in MPC.

Compressive strength was determined according to the method specified in Chinese national standard GB/T 17671-2021, and the samples that reached the correct age were loaded at a rate of 500 N/s using a microcomputer-controlled electro-hydraulic servo pressure testing machine (HCT-605A, Shenzhen Vance Testing Equipment Co., Ltd., Shenzhen, China). The main engine employed a high-performance bi-directional hydraulic cylinder loading with high reliability, zero leakage, and a maximum load of 3000 kN. After the samples were tested for strength, some blocks and some powders (grinding of blocks with an agate mortar) were taken for instrumental testing. Microstructure was analyzed by scanning electron microscopy (TM4000, Hitachi High-Technologies Corporation, Tokyo, Japan; acceleration voltage: 5 kV, 10 kV, 15 kV, 20 kV; sample movable range: X: 40 mm, Y: 35 mm; vacuum mode: standard and electrostatic reduction; image signal: backscattered electrons). The elemental composition of the micro-region was analyzed using energy-dispersive X-ray spectroscopy (EDS). The phase composition of the samples was analyzed by X-ray diffractometer (XRD) (DMAX1400, Smartlab, Rigaku, Japan, scanning speed 0.333° 2/s, scanning range 10–80°). Differential thermal analysis of MPC samples using a simultaneous thermal analyzer (STA8000, Perkin–Elmer, Waltham, MA, USA). The heating rate was 20 °C/min, the test atmosphere was N_2_, the gas flow rate was 50 mL/min, and the test temperature range was 30–300 °C.

The specimens’ pore size distribution and porosity were determined using the mercury-in-pressure (MIP) method with a poremaster device (33GT, Quantachrome Instruments, Boynton Beach, FL, USA). The maximum pressure was 33,000 Ib (228 MPa), and the aperture analysis range was 3 nm to ~360 µm.

## 3. Results and Analysis

### 3.1. Modification of MgO

**Figure 2:**
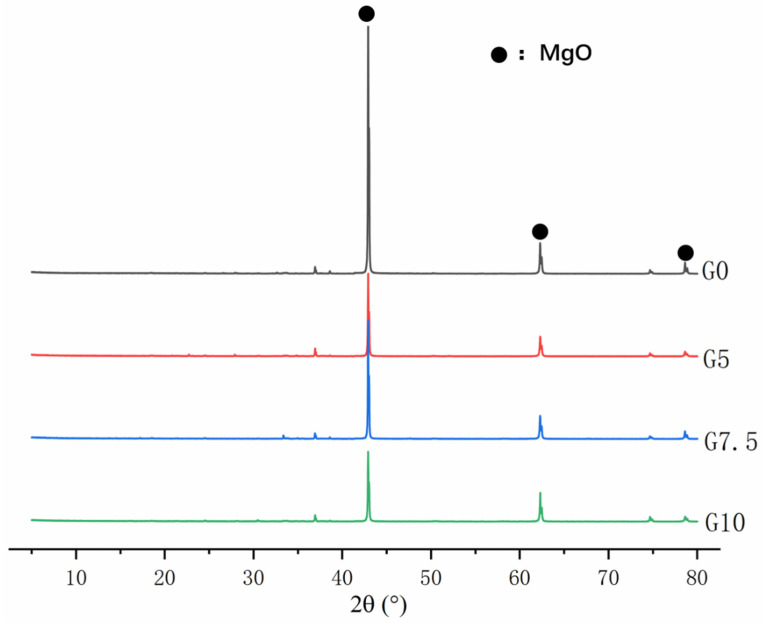
XRD patterns of water-glass-modified MgO with different doping levels.

**Figure 3:**
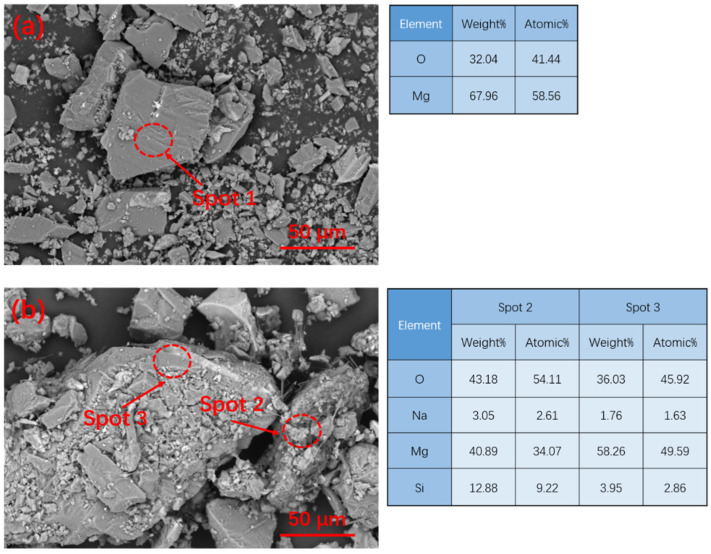
SEM pictures and micro-region composition of MgO before and after modification: (**a**) unmodified MgO; (**b**) modified MgO with 10% water glass.

Figure 2 shows the XRD patterns of MgO before and after the modification of MgO. As shown in Figure 2, it was found that the phase composition of MgO was still mainly MgO, and no new phase was generated. The microscopic morphology and micro-region composition of MgO are shown in Figure 3. The morphology of the MgO particles before and after modification is irregular and massive. Figure 3a shows that the surface of the unmodified MgO particles is relatively smooth, while Figure 3b shows that the modified MgO particles have a rough surface. According to the micro-region composition analysis results, a certain amount of silicon and sodium appeared on the surface, indicating that a coating layer was attached to the surface of MgO.

### 3.2. Working Performance

**Figure 4:**
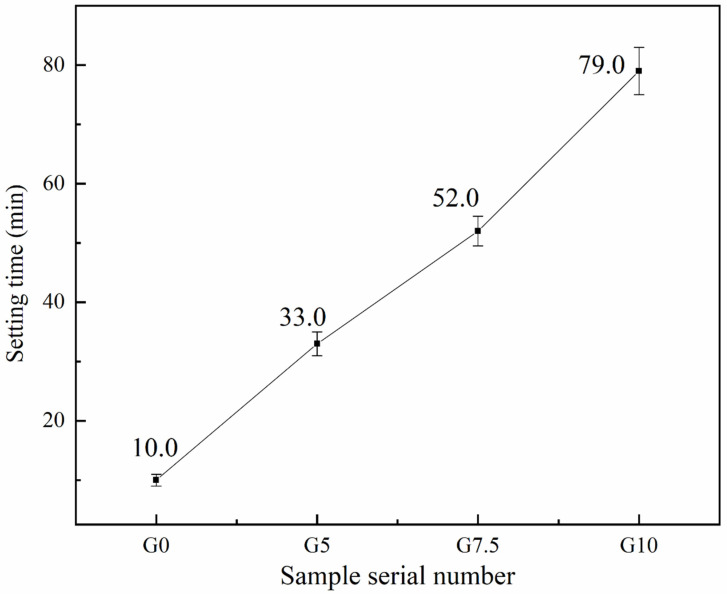
Effect of different water glass contents on the setting time of MPC at −5 °C.

Working performance, which is important for the application of magnesium phosphate cement, mainly includes fluidity and setting time. Figure 4 shows the change in the fluidity of MPC before and after modification at −5 °C. With the increase in sodium water glass content to 5%, 7.5%, and 10%, the setting times of MPC slurry can also rise to 33 min, 52 min, and 79 min, respectively, and the increase in each setting time reaches 230%, 420%, and 690%, respectively, compared with the reference group. This phenomenon indicates that the surface modification of magnesium oxide by sodium water glass causes a significant retarding effect in MPC.

**Figure 5:**
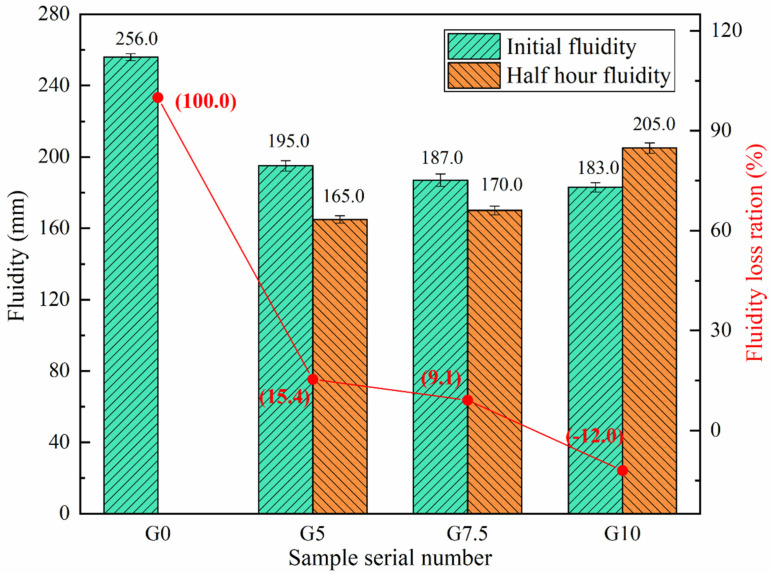
Effect of different water glass contents on the fluidity of MPC at −5 °C.

The effect of different water glass contents on the fluidity of MPC at −5 °C is shown in Figure 5. The results show that, although the unmodified MgO has a higher initial fluidity, there is no fluidity after 10 min due to a rapid setting time. Compared with the initial fluidity of 256 mm in the reference group, the fluidity of G5, G7.5, and G10 was reduced by 61 mm, 69 mm, and 73 mm, respectively. However, G5, G7.5, and G10 were still in a state of fluidity after 30 min, with fluidity loss rate of 15.4%, 9.1%, and −12%, respectively, as G10 showed an increase in fluidity at 30 min. This is because the water glass surface modification of MgO effectively reduces the dissolution rate of Mg^2+^, and the extension of the setting time makes it possible to maintain some fluidity after 30 min.

Through the analysis of the setting time and fluidity results, the surface modification of MgO by water glass can significantly increase these two important working properties and solve the issue of MPC hardening being too fast and difficult to construct under a negative-temperature environment.

### 3.3. Mechanical Properties

**Figure 6:**
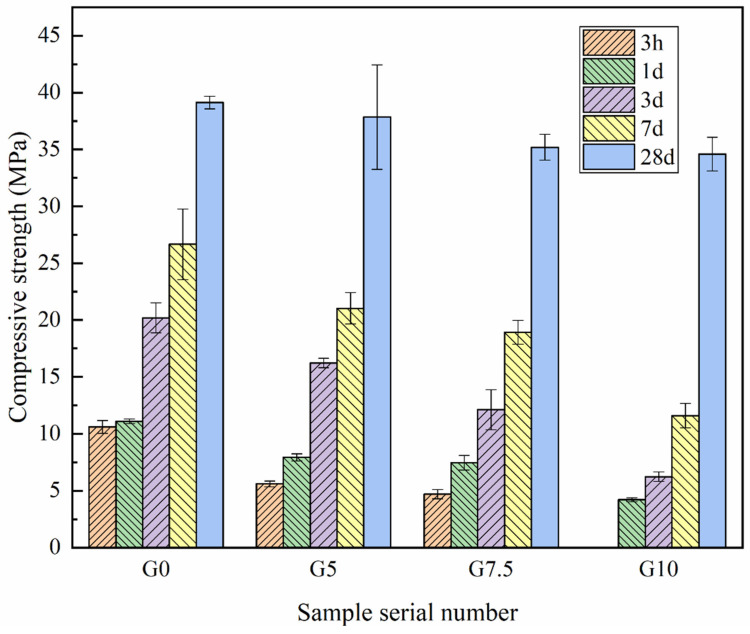
Effect of different water glass contents on compressive strength of MPC at −5 °C temperature.

Figure 6 shows the compressive strength of MPC modified with different sodium water glass doping at different curing ages at −5 °C. The results show that the compressive strength of each sample increases with the curing time. However, the compressive strength of the modified MgO is lower than that of the unmodified MgO. Its strength further decreases with the increase in sodium water glass, and this decrease in early strength is evident. Among them, the 3 hr compressive strength values of the samples with G5 and G7.5 were only 5.6 MPa and 4.7 MPa, 52.8% and 44.3% those of the unmodified MgO, and even when the sodium water glass addition was 10%, the 3 hr compressive strength was not significant. After 1 d of negative temperature curing, the compressive strengths of G5, G7.5, and G10 were 71.2%, 67.6%, and 37.8% those of the unmodified MgO, respectively, while the compressive strength of the unmodified components increased by only 4.7% from 3 h to 1 d. The compressive strengths of G0, G5, G7.5, and G10 were 39.1 MPa, 37.8 MPa, 35.2 MPa, and 34.6 MPa, respectively, after 28 d of curing, with some decrease in strength. The compressive strength of each sample increased from 1 d to 28 d by 257.0%, 379.7%, 369.3%, and 723.8%, respectively. This shows that MPC modified by sodium water glass can prolong the setting time and increase the growth rate of the later strength, and the 28 d strength of the maximum additions of the 10% sodium water glass all reached 88.5% of the unmodified MgO.

The above results show that the influence of modified MgO on strength development is mainly concentrated in the early stage of hydration. Compared with the unmodified sample, the strength of the sample with modified MgO is greatly reduced, but it can still achieve a specific strength. The effect of early strength is closely related to the setting time, and the surface modification of MgO slows down the process of hydration-product generation, which is the reason for the decrease in early strength. However, with the extension of the curing age, the strength increase in samples with modified MgO is more significant than that of the reference samples. When it reaches 28 days, the difference in strength is further reduced. This may be due to the gradual consumption of sodium silicate on the surface of MgO particles and the continuous reaction of the remaining phosphate with Mg^2+^.

### 3.4. Effect of Modified MgO on the Hydration Characteristics of MPC at Negative Temperature

**Figure 7:**
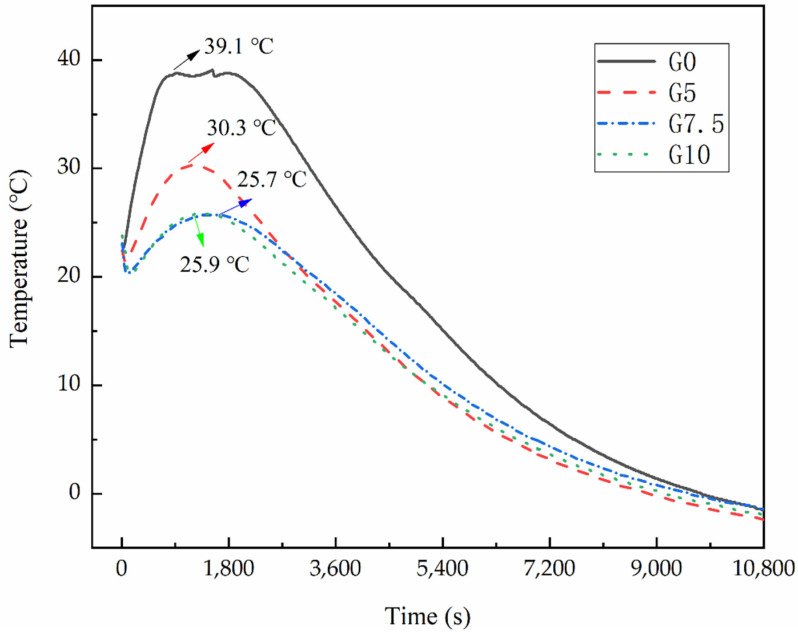
Effect of different water glass contents’ MgO on hydration temperature of MPC at −5 °C.

As the final setting time of MPC is completed within 3 Hr, its hydration heat release is also concentrated in this range. The hydration heat release is tested, and the results are shown in Figure 7. Figure 7 shows that the peak of hydration temperature of G5, G7.5, and G10 decreased by 22.51%, 34.27%, and 34.01% compared with that of G0, respectively, and the time to reach the peak was delayed by 3 min 40 s, 7 min 10 s, and 5 min 10 s, respectively. Meanwhile, the peak time of the reference group was as long as 15 min, while the modified components only maintained 2 min 10 s, 6 min 10 s, and 4 min 50 s, respectively. This is because the release rate of Mg^2+^ is slowed down by modification, which prolongs the formation time of hydration products and reduces the hydration exothermic heat, and the external ambient temperature of the matrix increases the rate of heat loss. Without modification, many Mg^2+^ ions quickly react with phosphate to form hydration products, so exothermic heat is quickly achieved, resulting in a sharp temperature rise. In addition, at the very first stage of the hydration reaction of MPC, the hydration temperature shows a trend of first decreasing and then increasing, except for the reference group. This is because the modification is made to slow down the release of Mg^2+^, while KDP and borax would absorb part of the heat when dissolved in water [28].

**Figure 8:**
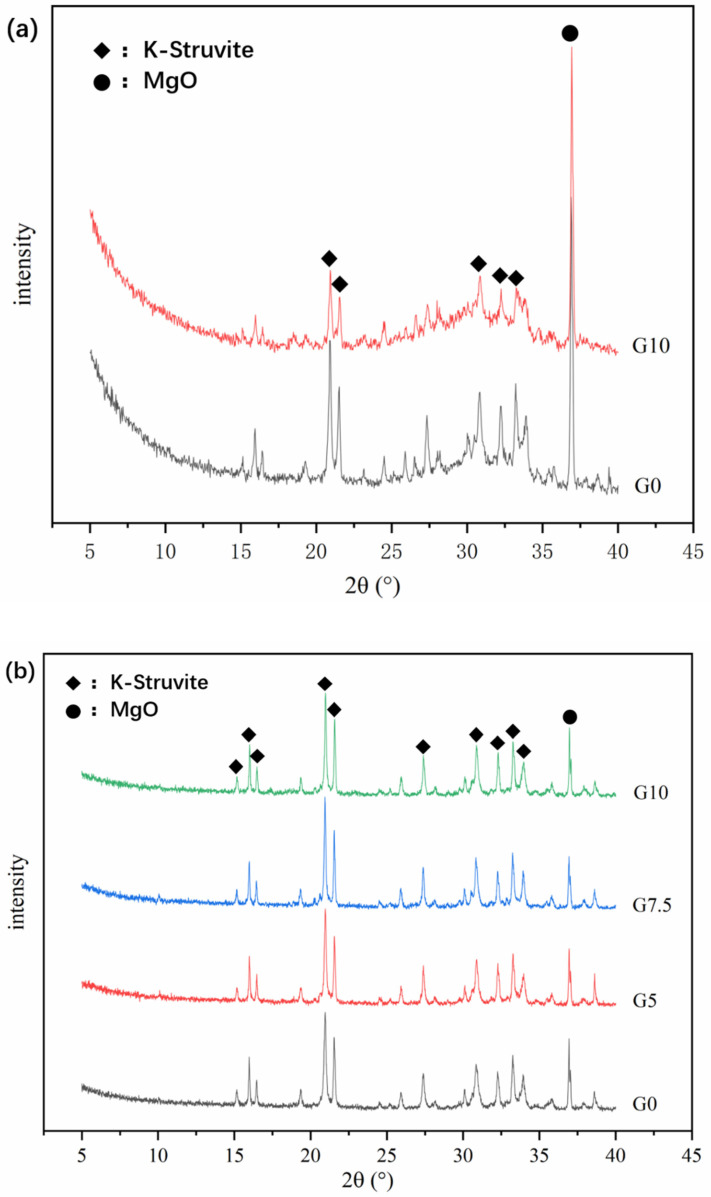
XRD patterns of MPC at different curing ages at −5 °C: (**a**) 3 h; (**b**) 28 d.

Figure 8 shows the XRD patterns of various modified MPCs’ curing at −5 °C. Figure 8a,b show the diffraction patterns of the sample after curing for 3 Hr and 28 d, respectively. The analysis shows that MgO before and after modification had no significant effect on the phase of the MPC’s early and final hydration products, and the main product was MgKPO_4_-6H_2_O. Furthermore, the crystalline phase of the G10-cured 3 Hr hydration product is clearly lower than that of G0. This further indicates that the surface modification of MgO has a higher effect on the early stage than on the later stage.

**Figure 9:**
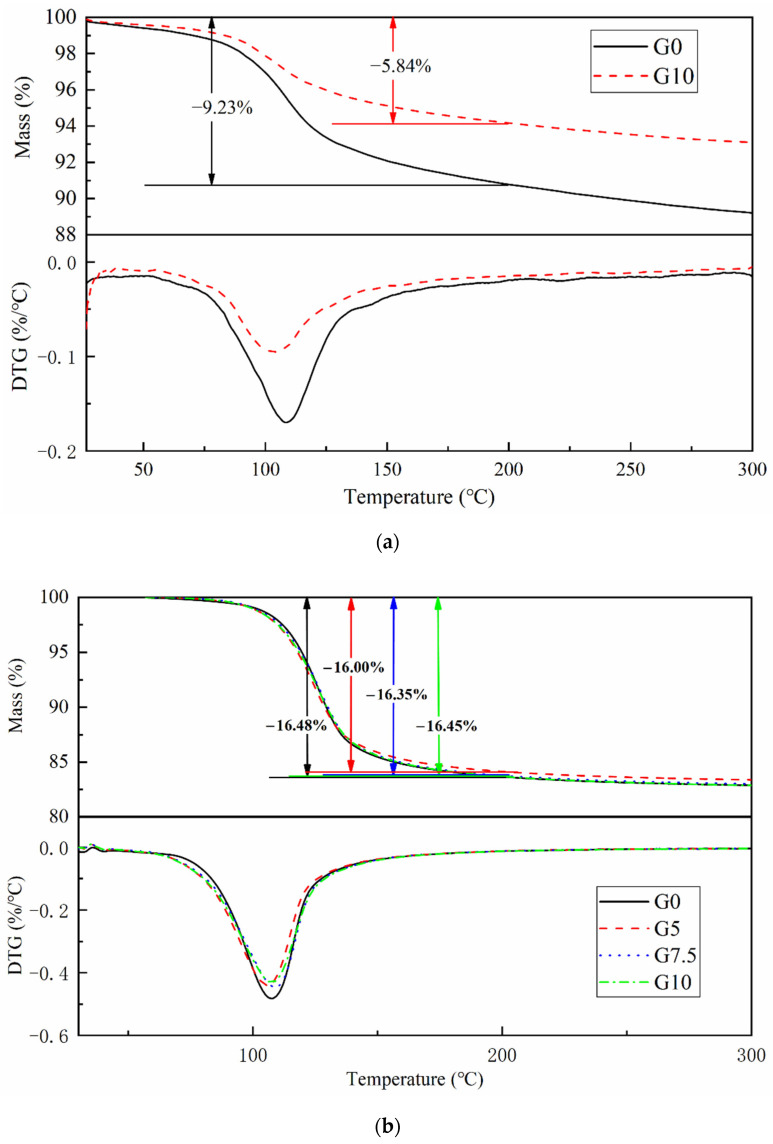
TG/DTG curves of MPC with various modified MgO at different curing ages: (**a**) 3 h; (**b**) 28 d.

Figure 9 shows the TG/DTG curves of various modified samples solidified at −5 °C. Figure 9a shows that the mass loss of G0 and G10 after curing for 3 Hr is 9.23% and 5.84%, respectively, which can be regarded as a large amount of hydration products because K-struvite completely decomposes from 30 °C to 200 °C [29,30]. This indicates that MgO surface modification greatly affects the early hydration of MPC, inhibiting the formation of hydration products, which is the combination of delaying the dissolution of Mg^2+^ and extending the setting time. Figure 9b shows that the mass losses of G0, G5, G7.5, and G10 at 200 °C after curing for 28 days are 16.48%, 16.00%, 16.35%, and 16.45%, respectively. The above results are consistent with the law of DTG peak area size. According to the analysis results, the surface modification of MgO has little effect on the final hydration products. The above results show that after 28 days of curing, the weight loss difference of all samples is small, which further indicates that the effect of modified MgO is mainly concentrated in the early stage of hydration and has no adverse effect on the strength development of the material in the later stage.

### 3.5. Effect of Modified MgO on the Microstructure of MPC at Negative Temperature

**Figure 10:**
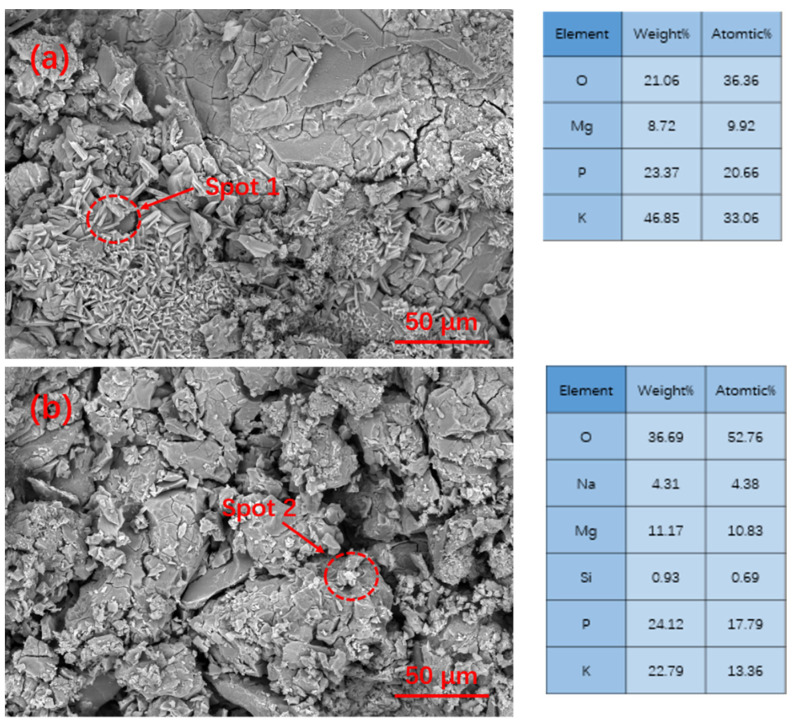
SEM/EDS pictures of MPC with various modified MgO cured at −5 °C for 3 h: (**a**) G0; (**b**) G10.

**Figure 11:**
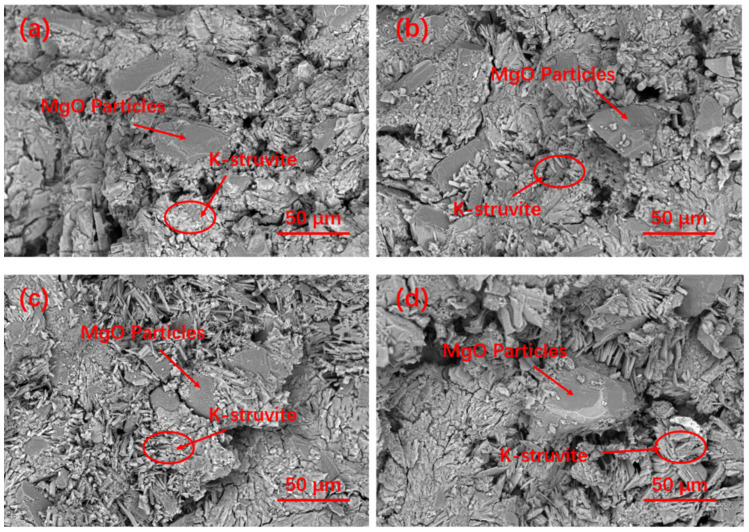
SEM pictures of MPC with various modified MgO cured at −5 °C for 28 d: (**a**) G0; (**b**) G5; (**c**) G7.5; (**d**) G10.

Figure 10 shows the microstructure of G0 and G10 after curing at −5 °C for 3 Hr. EDS was used to analyze the composition of their hydration products. It can be seen that the matrix structure of the reference group G0 in the early curing stage has become relatively dense, while the matrix structure of G10 is relatively loose. According to EDS analysis, K-struvite clearly formed on the MgO surface of the G0 matrix, while K-struvite was produced in G10 with a small amount and a large amount of phosphate. Figure 11a–d shows the SEM pictures of the sample after curing for 28 days. It can be seen that the K-struvite of the unmodified sample is mainly plate-shaped, and the K-struvite of the modified sample is significantly increased compared to that at the 3 Hr age, and there are many columnar K-struvite [31]. Moreover, this K-struvite grows around the surface of modified MgO and fills the gap between MgO particles, which is a reason for the rapid growth of the late strength of MPC. Although the amount of final hydration products of each modified sample in TG analysis is not that different, as shown in Figure 9, the columnar K-struvite is not as tight as the platelet structure, which makes the final strength of the modified MPC and unmodified MPC different.

**Figure 12:**
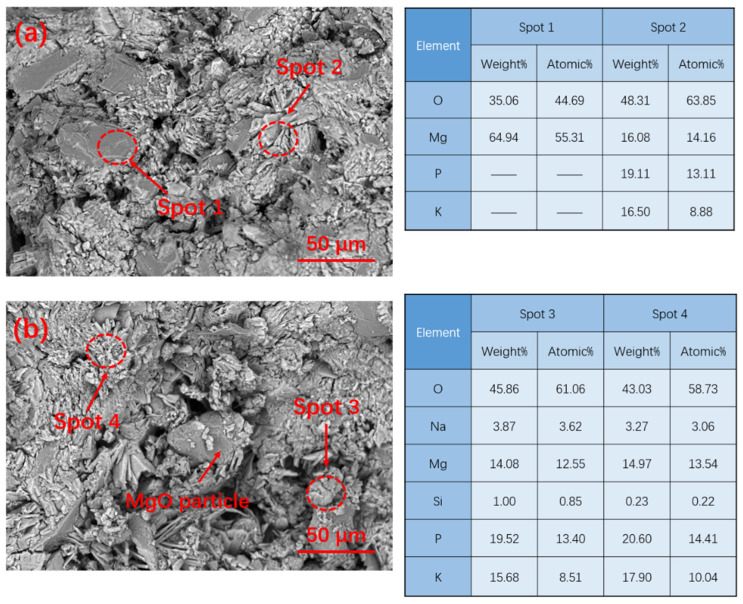
EDS pattern of MPC with various modified MgO curing at −5 °C for 28 d: (**a**) G0; (**b**) G10.

Figure 12 shows the EDS pattern analysis of MPC cured for 28 d. The EDS analysis shows that a certain amount of silicon and sodium can still be found on the surface of hydration products (Figure 11b), which shows that the high-temperature modification of sodium silicate has an obvious effect on reducing the activity of MgO.

### 3.6. Effect of Modified MgO on the Pore Structure of MPC at Negative Temperature

**Figure 13:**
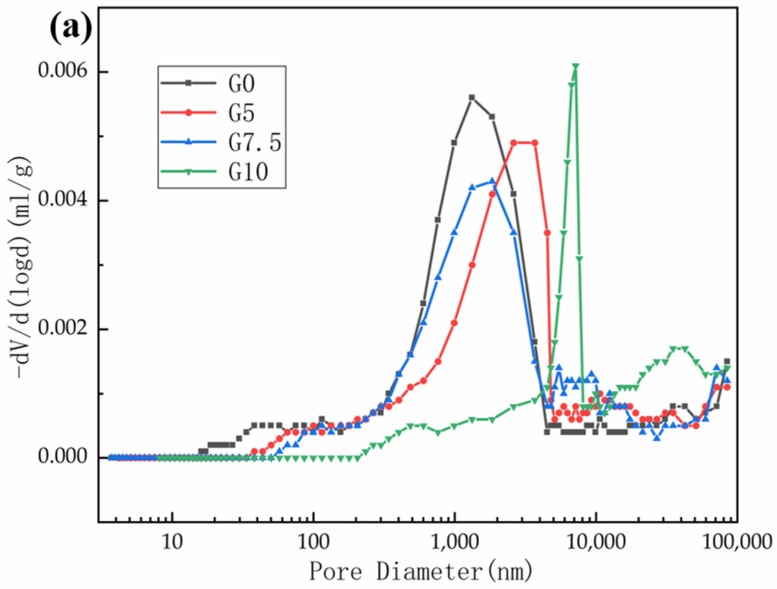

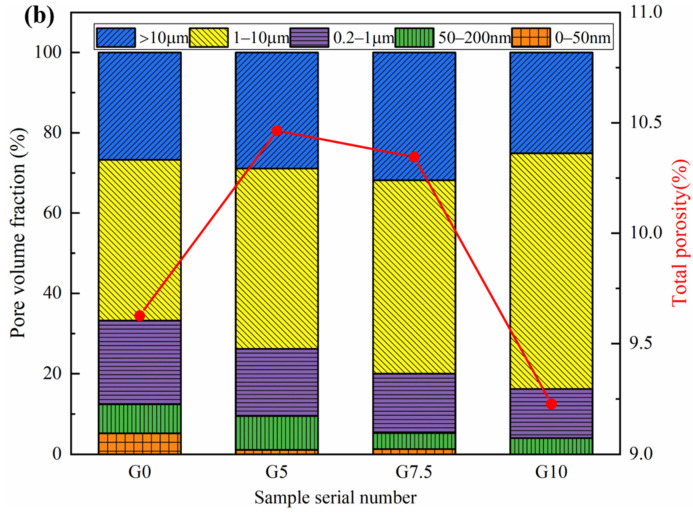
Effect of various modified MgO on the pore structure of MPC after 28 d curing at −5 °C: (**a**) pore size distribution; (**b**) pore volume fraction and total porosity.

After 28 d of curing, a mercury-pressure test was conducted to determine the relationship between the various amounts of modified MgO and the porosity of the MPC matrix. The results are shown in Figure 13. Figure 13a shows that, with the increase in the MgO-modification degree, the overall pore size of the sample increases, though the pore volume of pore size less than 1000 nm gradually decreased. When the pore size is larger than 1000 nm, its pore volume increases with the increase in the MgO-modification degree. However, when the pore size is larger than 10,000 nm, the pore volume of the sample modified with 10% water glass decreased (as seen in Figure 13b). Figure 13b shows that the total porosity of the sample tends to increase and then decrease with the increase in the MgO-modification degree, which may be due to the increase in the macropore ratio and the rapid growth of the hydration products at the late stage of MgO modification up to a certain degree, which makes the pores more filled. When the water glass modification reaches 10%, the total porosity is even lower than that of the reference group, which is only 9.23%. This indicates that with the increase in the MgO-modification degree, the fine pores within the matrix are gradually filled by K-struvite, allowing the matrix to develop strength later and at a higher growth rate than the reference group.

## 4. Conclusions

In this study, the hydration of MPC at a negative temperature (−5 °C) was investigated at different doping levels of sodium water glass, and its working properties, mechanical properties, and microstructure were analyzed. The obtained conclusions are as follows.

The surface modification of MgO effectively reduced the dissolution rate of Mg^2+^ and prolonged the coagulation time of MPC at −5 °C. When the amount of water glass modification reached 10%, the setting time increased from 10 min to 79 min without modification, equivalent to an increase of 690%.

Modified MgO can effectively improve the working performance of MPC. With a W/M ratio of 5−10%, MPC can control the fluidity loss rate to less than 20% after 30 min with an initial fluidity of 180 mm or more. When the modified amount reached 10%, the 30 min fluidity increased by 10%. The fluidity of the MPC pastes increases with the degree of MgO modification.

The MgO surface modification reduced the heat generated by MPC during the hardening process, while the effect of the negative-temperature environment caused the modified samples to have a reduced time to reach and sustain the peak heat of hydration.

When the W/M ratio was 10%, it made the MPC hydration early structure loose and the amount of hydration products decrease by one-third, which made the MPC early strength decrease. With the increase in W/M ratio and time, the amount of hydration product K-struvite filling the pores and cracks of the MPC matrix and the strength growth rate of MPC in the later stages of curing were both higher. When the W/M ratio was 10%, the late strength of MPC increased by 723.8%, and the matrix porosity was 9.23%, compared to 257.0% and 9.63% for the reference group, respectively. With little difference in the amount of final hydration products, the overall strength of the modified MPC matrix is reduced due to the poorer stability of columnar K-struvite compared to single slab.

An adequate setting time is good for practical applications under the condition of ensuring a good working performance. The early strength can be improved by using lightly burned MgO instead of dead-burned MgO, which, when compounded with phosphate, enables rapid use of restoration work.

## Figures and Tables

**Table 1:** Chemical composition of dead-burned magnesia (wt%).

MgO	SiO_2_	CaO	P_2_O_5_	Fe_2_O_3_	Al_2_O_3_	Others
95.12	1.76	1.04	0.83	0.56	0.49	0.2

**Table 2:** Raw material modification and sample mix design.

Series	W/M Ratio (wt%)	Thermal Treatment Temperature	Thermal Treatment Time	M/P ^a^	B/M ^b^	*w*/*s* ^c^
G0	0	—	—			
G5	5	150 °C	2 h	3:1	0.1	0.2
G7.5	7.5					
G10	10					

Note: W—water glass; M—MgO; P—KH_2_PO_4_; B—borax; w—water (0 °C); s—solid; ^a^ M/P is the MgO–KH_2_PO_4_ mass ratio; ^b^ B/M is the borax–MgO mass ratio; ^c^ *w*/*s* is the water–solid mass ratio.

## Data Availability

Data can be provided by contacting the author.
